# *MIR17HG* polymorphisms contribute to high-altitude pulmonary edema susceptibility in the Chinese population

**DOI:** 10.1038/s41598-022-06944-8

**Published:** 2022-03-14

**Authors:** Lining Si, Haiyang Wang, Yahui Jiang, Yun Yi, Rong Wang, Qifu Long, Yanli Zhao

**Affiliations:** 1 Department of Critical-Care Medicine, Affiliated Hospital of Qinghai University, Xining, 810001 Qinghai China; 2 Medical College, Qinghai University, No. 29 Tongren Road, Xining, 810001 Qinghai China; 3 Department of Diabetes of Traditional Chinese Medicine, Qinghai Red Cross Hospital, Xining, 810001 Qinghai China

**Keywords:** Genetics, Risk factors

## Abstract

High-altitude pulmonary edema (HAPE) is a common acute altitude sickness. This study was designed to investigate the effect of *MIR17HG* polymorphisms on HAPE risk in the Chinese population. The Agena MassARRAY platform was used to genotype six single-nucleotide polymorphisms (SNPs) in the *MIR17HG* gene in 244 HAPE patients and 243 non-HAPE controls. The odds ratio (OR) and 95% confidence interval were used to evaluate the association between each *MIR17HG* polymorphisms and the risk of HAPE under a polygenetic model. Statistical analysis was performed using the χ^2^ test. Multifactor dimensionality reduction (MDR) analysis was used to analyze the impacts of SNP–SNP interactions on the risk of HAPE. According to the allele model, the HAPE risk of people with the rs7318578 A allele of *MIR17HG* was lower than that of people with the C allele (OR 0.74, *p* = 0.036).Logistic regression analysis of four models for all selected *MIR17HG* SNPs showed significant differences in the frequencies of rs7318578 (OR 0.74, *p* = 0.037) and rs17735387 (OR 1.51, *p* = 0.036) between cases and controls. The results of the sex stratification analysis showed that among males, rs17735387 in the *MIR17HG* gene is associated with an increased risk of HAPE. *MDR* analysis showed that the best combination model was a three-locus model incorporating rs72640334, rs7318578, and rs7336610. This study revealed the correlations between rs7318578 and rs17735387 on the *MIR17HG* gene and the risk of HAPE in the Chinese population, providing a theoretical basis for the early screening, prevention, and diagnosis of HAPE in high-risk populations.

## Introduction

Among plateau-related diseases, acute mountain sickness (AMS) is the most common. There are regional differences in the incidence of AMS. Generally, the higher the altitude is, the higher the incidence of AMS and the more severe the clinical symptoms. AMS includes two types: mild and severe. High-altitude pulmonary edema (HAPE) is a severe form of AMS^1^. This diseaseis a serious life-threatening noncardiogenic pulmonary edema caused by a high-altitude hypoxic environment. HAPE is a special and common disease that develops after a short period of exposure to altitudes above 2250 m and is characterized by acute onset, rapid progress and serious consequences^1,2^. People living in the plains usually have HAPE within 2 to 5 days after entering the plateau. Specifically, when people are rapidly exposed to high-altitude environments, low oxygen air tension can cause acute pulmonary artery vasoconstriction, which in turn leads to an increase in high pulmonary artery pressure. The resulting pressure on the pulmonary microvascular system can cause excessive fluid to leak into the lungs, and ultimately lead to the occurrence of HAPE^3^. It can be seen that the high-altitude and low-oxygen environment easily leads to the occurrence of HAPE. Increasing evidence has shown that genetic factors, such as IL1R2^4^, NR3C2^5^, NR3C1^6^, and NOS3^7^, also play key roles in the occurrence of HAPE.

The pathogenesis of HAPE is more complex, and it is unclear. It is generally believed that the key to its occurrence is the pulmonary vasoconstriction and increased pulmonary arterial pressure caused by hypoxia at high altitude. Studies have found that lncRNA polymorphisms were associated with genetic susceptibility to pulmonary hypertension^8^. Therefore, we believe that lncRNA polymorphisms may also be associated with genetic susceptibility to high altitude pulmonary edema.

*MIR17HG* is derived from the mir-17-92a-1 cluster host gene and is a class of pri-miRNAs located in the 800 base pair region of human chromosome 13. Studies have confirmed that *MIR17HG* plays important roles in various growth and developmental processes, such as cell proliferation and differentiation and angiogenesis^9–11^. Wei et al.^12^ evaluated the expression of *MIR17HG* is down-regulated in NSCLC, while the over-expression of *MIR17HG* can lead to up-regulation of miR-142-3p and down-regulation of Bach-1 expression, thereby inhibiting the invasion and migration ability of non-small cell lung cancer (NSCLC) cells through methylation-specific PCR (MSP) and Transwell invasion and migration tests. By conducting an experiment with a mouse model, Rogers et al.^13^ showed that the expression of *MIR17HG* is related to bronchopulmonary dysplasia. Liao et al.^14^ analyzed asthma-related information from an online database and showed that *MIR17HG* plays an important role in asthma and may become a potential target and prognostic biomarker for asthma treatment. These findings indicate that *MIR17HG* plays an important role in lung diseases. A number of studies have shown that *MIR17HG* polymorphisms are associated with various disease risks^15^, and multiple reports have shown that gene polymorphisms are associated with HAPE risk^16^. However, there is no report on the relationship between *MIR17HG* polymorphisms and HAPE risk.. This study aims to explore the correlations between *MIR17HG* polymorphisms and the risk of HAPE in the Chinese population through a case–control strategy to provide a theoretical basis for the early screening, prevention and diagnosis of HAPE in high-risk groups.

## Results

### Basic characteristics of the participants

As Table 1 shows, 487 participants were recruited. Among them, 244 participants had HAPE (19 females, 225 males), while 243 participants had no symptoms of HAPE (19 females, 224 males). The mean ages in the HAPE group and healthy group were 32.41 ± 10.81 and 32.70 ± 9.52 years, respectively. No significant differences were found in sex or age between the case and control groups (both *p* > 0.05). The selected SNPs were functionally annotated using HaploRegv4.1 software. The results showed that these six SNPs (rs75267932, rs72640334, rs7336610, rs7318578, rs17735387 and rs1428) in the *MIR17HG* gene are associated with promoter histone marks, enhancer histone marks, DNase, motif changes, and GRASP QTLs, suggesting that they may play important roles in related biological functions in patients.

**Table 1:** Characteristics of participants.

Variables	Case (244)	Control (243)	*p*
Age ± SD	32.41 ± 10.81	32.70 ± 9.52	0.761
**Sex**			0.869
Female	19	19	
Male	225	224	

*p* < 0.05 indicates statistical significance.

### Associations of genetic polymorphisms with HAPE risk

Table 2 shows the basic information of the six SNPs in the *MIR17HG* gene identified in this study. All SNPs were found to be at Hardy–Weinberg equilibrium (HWE) based on exact tests. In the allele model, we observed a lower risk of HAPE (odds ratio (OR) 0.74, 95% confidence interval (95% CI) 0.56–0.98, *p* = 0.036) associated with the A allele of *MIR17HG* SNP rs7318578 than with the C allele. Logistic regression analysis of the four models for all of the selected *MIR17HG* SNPs between cases and controls showed significant differences for rs7318578 and rs17735387 in the HAPE population. For rs7318578, under the log-additive model, we found that people carrying the A allele had a decreased risk of HAPE compared with C allele carriers, which with the A allele playing a protective role (OR 0.74, 95% CI 0.56–0.98, *p* = 0.037; Table 3). Individuals carrying the rs17735387 A allele had a 51% higher risk of HAPE than those with the G allele (OR 1.51, 95% CI 1.03–2.21, *p* = 0.036; Table 3).

**Table 2:** Basic information of six SNPs on the *MIR17HG* gene in this study.

SNP	Chromosome	BP	Alleles	Gene	MAF-Case	MAF-Control	HWE-*p*	OR (95% CI)	*p*	HaploReg
rs75267932	13	91,351,812	G/A	*MIR17HG*	0.125	0.115	0.75	1.10 (0.75–1.62)	0.639	Promoter histone marks, Enhancer histone marks, DNAse, Motifs changed
rs72640334	13	91,352,674	A/C	*MIR17HG*	0.102	0.103	0.726	1.00 (0.66–1.51)	0.983	Enhancer histone marks, DNAse Motifs changed
rs7336610	13	91,352,883	C/T	*MIR17HG*	0.471	0.484	0.608	0.95 (0.74–1.22)	0.703	Enhancer histone marks, Proteins bound, Motifs changed, Selected eQTL hits
rs7318578	13	91,353,215	A/C	*MIR17HG*	0.256	0.317	0.882	0.74 (0.56–0.98)	**0.036**	Enhancer histone marks, DNAse, Motifs changed, GRASP QTL
rs17735387	13	91,353,800	A/G	*MIR17HG*	0.199	0.16	0.229	1.30 (0.93–1.80)	0.12	Enhancer histone marks, DNAse, Proteins bound, Motifs changed
rs1428	13	91,354,516	C/A	*MIR17HG*	0.475	0.484	0.608	0.97 (0.75–1.25)	0.8	Enhancer histone marks, DNAse, Proteins bound, Motifs changed

*HWE* Hardy–Weinberg equilibrium, *ORs* odds ratios, *CI* confidence interval.

*p* < 0.05 indicates statistical significance.

Significant values are in bold.

**Table 3:** Correlation of *MIR17HG* SNPs with HAPE risk.

SNP	Model	Genotype	Number		Crude analysis		adjusted by age and sex	
			Case	Control	OR (95% CI)	*p*	OR (95% CI)	*p*
rs7318578	Genotype	AA	134	114	1		1	
		AC	95	104	0.78 (0.53–1.13)	0.186	0.78 (0.54–1.14)	0.198
		CC	15	25	0.51 (0.26–1.02)	0.055	0.51 (0.26–1.01)	0.053
	Dominant	CC/AC + AA	15/229	25/218	0.73 (0.51–1.04)	0.078	0.73 (0.51–1.04)	0.081
	Recessive	CC + AC/AA	110/134	129/114	0.57 (0.29–1.11)	0.1	0.57 (0.29–1.10)	0.094
	Log-additive	–	–	–	0.74 (0.56–0.98)	0.037	0.74 (0.56–0.98)	**0.037**
rs17735387	Genotype	GG	153	174	1		1	
		AG	85	60	1.61 (1.09–2.39)	0.018	1.62 (1.09–2.41)	**0.018**
		AA	6	9	0.76 (0.26–2.18)	0.607	0.76 (0.27–2.20)	0.617
	Dominant	AA/AG + GG	6/238	91,377/234	1.50 (1.03–2.20)	0.037	1.51 (1.03–2.21)	**0.036**
	Recessive	AA + AG/GG	91/153	69/174	0.66 (0.23–1.87)	0.43	0.66 (0.23–1.88)	0.434
	Log-additive	–	–	–	1.30 (0.93–1.81)	0.119	1.31 (0.94–1.82)	0.117

*SNP* single-nucleotide polymorphism, *OR* odds ratio, *95% CI* 95% confidence interval.

Significant values are in bold.

### Stratification analysis of the associations of SNPs with HAPE risk

To further clarify the associations between *MIR17HG* polymorphisms and HAPE risk, we conducted age- and sex-stratified analyses. In the age stratification analysis, we found no association of any of the six polymorphic loci (rs75267932, rs72640334, rs7336610, rs7318578, rs17735387, and rs1428) in the *MIR17HG* gene with the risk of HAPE (Supplementary Table 2). The results of the sex stratification analysis showed that among men, individuals with the rs17735387 genotype AG had an increased risk of HAPE compared with GG individuals (OR 1.55, 95% CI 1.03–2.32, *p* = 0.036; Table 4).

**Table 4:** Stratification analysis by gender for the effect of *MIR17HG* SNPs on HAPE risk.

SNP	Model	Genotype	Male	*p*
			OR (95% CI)	
rs75267932	Genotype	AA	1	
		AG	1.19 (0.76–1.85)	0.455
		GG	1.04 (0.14–7.44)	0.972
	Dominant	GG/AG + AA	1.18 (0.76–1.83)	0.462
	Recessive	GG + AG/AA	1.00 (0.14–7.14)	0.997
	Log-additive	–	1.16 (0.77–1.75)	0.488
rs72640334	Genotype	CC	1	
		AC	0.88 (0.55–1.43)	0.616
		AA	1.43 (0.24–8.73)	0.697
	Dominant	AA/AC + CC	0.91 (0.57–1.45)	0.688
	Recessive	AA + AC/CC	1.47 (0.24–8.92)	0.677
	Log-additive	–	0.94 (0.61–1.45)	0.790
rs7336610	Genotype	TT	1	
		TC	1.47 (0.94–2.32)	0.094
		CC	0.87 (0.51–1.51)	0.624
	Dominant	CC/TC + TT	1.26 (0.82–1.94)	0.294
	Recessive	CC + TC/TT	0.67 (0.43–1.05)	0.084
	Log-additive	–	0.95 (0.72–1.24)	0.697
rs7318578	Genotype	AA	1	
		AC	0.79 (0.53–1.17)	0.232
		CC	0.53 (0.27–1.07)	0.076
	Dominant	CC/AC + AA	0.73 (0.51–1.07)	0.104
	Recessive	CC + AC/AA	0.59 (0.3–1.16)	0.126
	Log-additive	–	0.75 (0.56–1.01)	0.055
rs17735387	Genotype	GG	1	
		AG	1.55 (1.03–2.32)	**0.036**
		AA	0.63 (0.2–1.91)	0.412
	Dominant	AA/AG + GG	1.42 (0.96–2.11)	0.077
	Recessive	AA + AG/GG	0.54 (0.18–1.65)	0.283
	Log-additive	–	1.23 (0.88–1.73)	0.233
rs1428	Genotype	AA	1	
		AC	1.4 (0.89–2.2)	0.147
		CC	0.92 (0.53–1.57)	0.748
	Dominant	CC/AC + AA	1.23 (0.8–1.88)	0.350
	Recessive	CC + AC/AA	0.73 (0.47–1.14)	0.168
	Log-additive	–	0.97 (0.74–1.26)	0.802

*SNP* single nucleotide polymorphism, *OR* odds ratio, *95% CI* 95% confidence interval.

Bold indicated that *p* < 0.05 meant the data was statistically significant.

### SNP–SNP interactions

**Figure 1:**
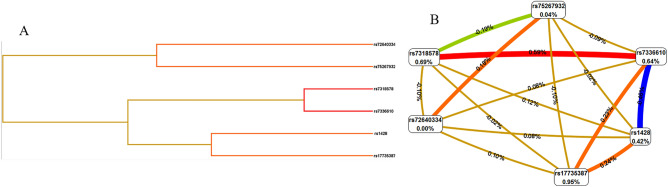
*MDR* analysis results. *MDR*-3.0.2 software: http://sourceforge.net/projects/mdr/. Dendogram (**A**) and Fruchterman Rheingold plot (**B**) of the interactions of *MIR17HG* SNPs rs75267932, rs72640334, rs7336610, rs7318578, rs17735387 and rs1428 for HAPE risk. Shorter connections among nodes indicate stronger redundant interactions. Negative percent entropy indicates redundancy.

As shown in Fig. 1A, B a dendrogram and a Fruchterman Reingold algorithm were used to describe the interactions among these SNPs. We evaluated all three-way to five-way models (Table 5). We chose a three-loci model as the best model considering the small sample size. The overall best model included rs72640334, rs7318578, and rs7336610, with a testing accuracy of 56.58%, a maximum cross-validation consistency (CVC) of 10/10 and p value of < 0.001 obtained by the χ^2^ test. Compared with the low-risk genotype combination, the generated combination of high-risk genotypes was associated with an increased HAPE risk (OR 2.43, 95% CI: 1.61–3.68, *p* < 0.001; Table 5). The MDR approach uses entropy to evaluate the correlation between variable combinations and attribute interactions (IG)^17^. The strongest interaction effect was found between rs7318578 and rs7336610, with an IG value of 0.59%.

**Table 5:** SNP–SNP interaction models of *MIR17HG* gene the predisposition of HAPE.

Model	Testing Bal. Acc. (%)	CVC	OR (95% CI)	*p*
rs17735387	51.44	7/10	1.64 (1.11–2.43)	**0.013**
rs7318578,rs7336610	53.09	9/10	2.26 (1.43–3.57)	**< 0.001**
rs72640334,rs7318578,rs7336610	56.58	10/10	2.43 (1.61–3.68)	**< 0.001**
rs72640334,rs7318578,rs7336610,rs75267932	53.09	8/10	2.34 (1.58–3.46)	**< 0.001**
rs1428,rs17735387,rs72640334,rs7318578,rs75267932	52.67	6/10	2.39 (1.61–3.55	**< 0.001**
rs1428,rs17735387,rs72640334,rs7318578,rs7336610,rs75267932	52.88	10/10	2.34 (1.57–3.49)	**< 0.001**

*MDR* multifactor dimensionality reduction, *Bal. Acc.* balanced accuracy, *CVC* cross-validation consistency.

*p* values were calculated using χ^2^ test.

Bold indicated that *p* < 0.05 means the data is statistically significant.

### Haplotype analysis with the risk of HAPE

**Figure 2:**
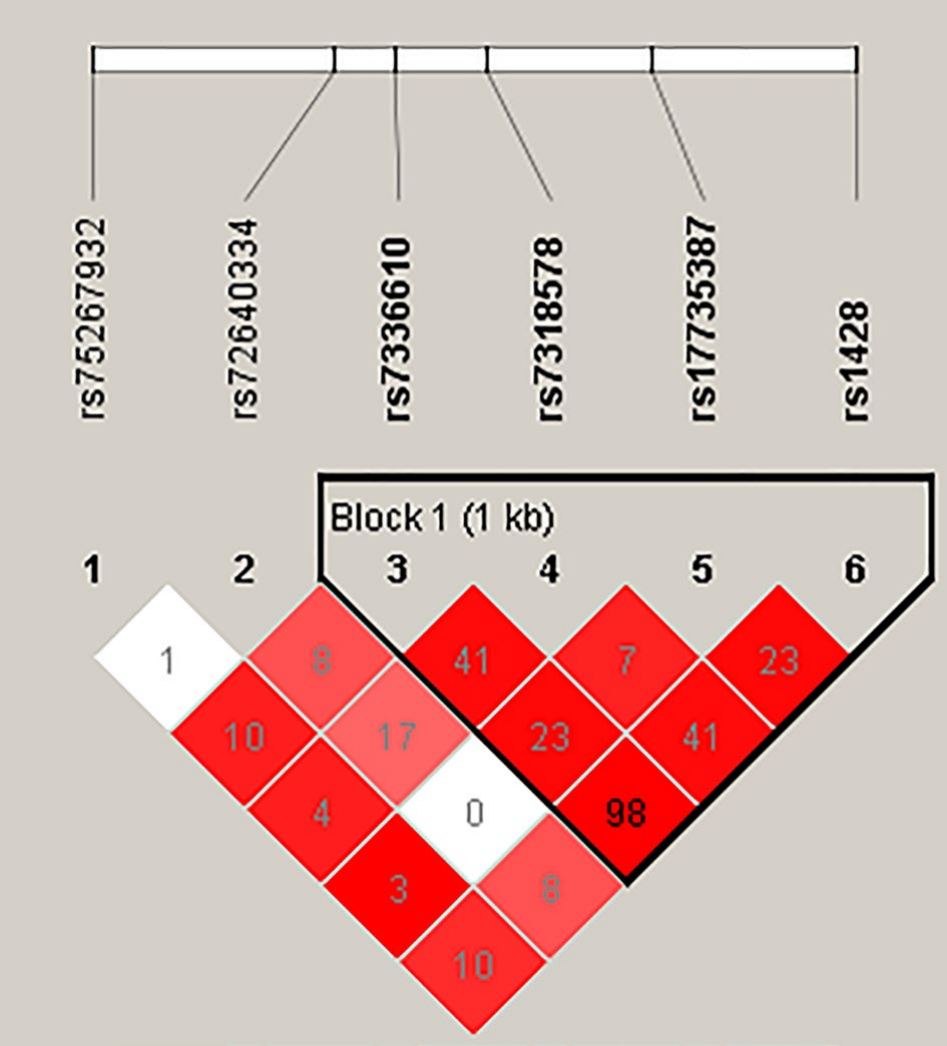
LD plots of six SNPs in the *MIR17HG* gene. Haploview-4.2 software: https://www.broadinstitute.org/haploview/downloads#JAR. The squares in the graph represent the strength of linkage disequilibrium (r2) between every two SNPs. The darker the color, the greater the r2 value. The inverted black triangles represent haplotype blocks defined by the confidence interval method. Agena MassARRAY Assay Design 3.0 software: https://seqpws1.agenabio.com/AssayDesignerSuite.html#design:new=Genotyping. Agena Typer 4.0 software: (Agena, San Diego, CA, USA) https://seqpws1.agenabio.com/AssayDesignerSuite.html#design:new=Genotyping. The Agena Typer 4.0 software for genotyping was purchased from the MassARRAY chip company (Agena, San Diego, CA, USA).

In addition, we conducted linkage disequilibrium (LD) and haplotype analysis of *MIR17HG* polymorphisms. The constructed LD map is shown in Fig. 2. The LD block consists of four SNPs, rs7336610, rs7318578, rs17735387 and rs1428, in the *MIR17HG* gene. The frequency distributions of the haplotypes in the case group and the control group are shown in Table 6. However, there was no significant association between haplotype and HAPE risk.

**Table 6:** The haplotype analysis result of *MIR17HG* polymorphisms and their associations with HAPE risk.

Gene	SNP	Haplotype	Frequency		Without adjusted		Adjusted	
			HAPE patients	Control	OR (95% CI)	*p*	OR (95% CI)	*p*
*MIR17HG*	rs7336610|rs7318578|rs17735387|rs1428	CAAC	0.803	0.844	0.76 (0.54–1.05)	0.099	0.75 (0.54–1.05)	0.098
*MIR17HG*	rs7336610|rs7318578|rs17735387|rs1428	CCGC	0.746	0.691	1.33 (0.99–1.77)	0.055	1.32 (0.99–1.77)	0.055
*MIR17HG*	rs7336610|rs7318578|rs17735387|rs1428	CAGC	0.982	0.986	0.77 (0.28–2.11)	0.618	0.78 (0.28–2.12)	0.622
*MIR17HG*	rs7336610|rs7318578|rs17735387|rs1428	TAGA	0.518	0.512	1.03 (0.79–1.33)	0.846	1.03 (0.79–1.33)	0.85

*SNP* single-nucleotide polymorphism, *OR* odds ratio, *95% CI* 95% confidence interval.

*p* value was calculated by Wald test with and without adjusted by age and gender. *p* < 0.05 means the data is statistically significant.

### Meta-analysis

Correlation analysis was carried out on the genotyping data of six SNPs on *MIR17HG* of HAPE that has been reported. Meta analysis showed that rs7318578 on the *MIR17HG* gene reduced the risk of HAPE under the allele model (OR 0.77, 95% CI 0.61–0.98, *p* = 0.037) (Supplementary Table 3), which is consistent with our previous research results.

## Discussion

HAPE is a very harmful disease to humans. This study explored the impact of the MIR17HG polymorphisms on the risk of HAPE in the Chinese population. In our study, we found that *MIR17HG* rs7318578 minimized the risk and that rs17735387 increased the risk.

*MIR17HG* is a noncoding RNA that affects protein synthesis by regulating gene expression, and its sole function is to produce six miRNAs (miR-17, miR-18a, miR-19a, miR-20a, miR-19b-1, and miR-92-1)^18,19^. Yin et al.^20^ showed that miR-17 is involved in the regulation of EGFR-TKI resistance and can be used as a predictive biomarker for NSCLC EGFR-TKI resistance. Li et al.^21^ showed that the expression of miR-18a affects the occurrence of lung fibroblast fibrosis. In addition, Ren et al.^22^ showed that the downregulation of miR-19a-3p expression is related to the reduction of sepsis-induced lung injury. These findings indicate that *MIR17HG* plays an important role in the occurrence and development of a variety of lung diseases. Multiple reports have revealed that *MIR17HG* polymorphisms are associated with various disease risks^23,24^, however, there is no report about the polymorphisms of *MIR17HG* and the risk of HAPE.. Our research revealed that people carrying the rs7318578 A allele had a reduced risk of HAPE compared with C allele carriers, and that individuals carrying the rs17735387 A allele had a 51% increased risk of HAPE compared with G-allele carriers. Meta-analysis also showed that rs7318578 is related to the reduction of HAPE risk. Our study further revealed that the *MIR17HG* polymorphisms are associated with the risk of HAPE. The incidence of HAPE differs between the sexes. The results of the sex stratification analysis showed that among males, individuals with the rs17735387 AG genotype had an increased risk of HAPE compared with GG individuals, which indicated that the rs17735387 polymorphism of the *MIR17HG* gene affects the risk of HAPE. This result has implications for the screening of biomarkers of HAPE risk in male populations.

Studies have confirmed that p53 and HIF-1α can inhibit the expression of *MIR17HG*^25^. In addition, studies have shown that VEGF-induced *MIR17HG* expression is involved in the regulation of angiogenesis^26^. Chamorro-Jorganes et al.^27^ showed that the downstream effector of HIF-1α, VEGF, affects the occurrence of HAPE by regulating vascular permeability. However, there is no report on the association between *MIR17HG* and HAPE, and we hypothesize that there is a correlation between the two. The promoter of the miR-17-92 cluster host gene *MIR17HG* is highly conserved, and the promoter region has multiple conserved transcription factor binding sites^28^.SNPs might impact biological processes involved in the production or functional effects of the miR-17-92 cluster host gene. We found that *MIR17HG* rs7318578 minimized the risk of HAPE and that rs17735387 increased the risk. These results provide some empirical support for our hypothesis, however, to clarify the associations between *MIR17HG* and HAPE, we conducted an MDR analysis. MDR is a recently developed method for analyzing interactions. It can simultaneously detect and describe the effects of multifactor interactions on diseases and has been widely used in scientific research^29,30^. This study clarified the influences of *MIR17HG* polymorphism site interactions on the risk of HAPE through MDR. The results showed that rs7318578 and rs7336610 had a strong synergistic effect on HAPE sensitivity and that rs7336610 and rs1428 had a strong antagonistic effect on HAPE sensitivity (Fig. 1B). The best model combination for HAPE sensitivity prediction was rs72640334, rs7318578, and rs7336610. All of these results upport our hypothesis, which *MIR17HG* polymorphisms are related to HAPE.

This study has limitations. First, the sample size was small. Second, although the correlation between six SNPs in *MIR17HG* and the risk of HAPE in the Chinese population was explored in this study, the *MIR17HG* gene includes many other sites. In our future work, we will explore the correlations between other sites on *MIR17HG* and the risk of HAPE. Third, due to a lack of cell biology methods, we did not explore the molecular mechanisms underlying the influences of *MIR17HG* polymorphisms on the occurrence of HAPE. In the future, we will explore the specific role of *MIR17HG* in HAPE from these perspectives.

In conclusion, this study clarified that *MIR17HG* polymorphisms are associated with the risk of HAPE in the Chinese population, which lays a foundation for further research on the mechanism of *MIR17HG* action on HAPE and provides a scientific basis for early screening and prevention of HAPE in high-risk population.

## Methods

### Participants

Based on the case–control strategy for exploring polymorphisms in the *MIR17HG* gene, we recruited 487 participants from the Affiliated Hospital of Qinghai University. All participants lived in low-altitude areas below 2500 m and reached high-altitude areas above 3000 m due to work or travel. By examining the symptoms (dyspnea), signs (cyanosis at rest) and imaging results (such as X-ray radiography and computed tomography (CT) of the patient chest) for all participants, and adhering to with the diagnostic criteria of HAPE^31^, we found that 244 participants had HAPE, forming the case group, while 243 participants had no symptoms of HAPE, forming the control group. There were 225 males and 19 females in the case group, and 224 and 19 females in the control group.

### Genotyping

To ensure the successful statistical analysis of genotypes in the research population, SNPs with a minor allele frequency of at least 5% in the Han Chinese from Beijing (CHB) population were initially screened using the Thousand Genomes Project database (http://www.internationalgenome.org/). Haploview software (version 4.2) (https://www.broadinstitute.org/haploview/downloads#JAR) was then used to conduct LD haplotype analysis^32^. Subsequently, function prediction analysis (https://www.ncbi.nlm.nih.gov/snp/) of tag single nucleotide polymorphisms (tagSNP) was performed online with the dbSNP database and showed that the tagSNPs are introns that affect the activity of splice sites. Following further analysis, six SNPs in the *MIR17HG* gene (rs75267932, rs72640334, rs7336610, rs7318578, rs17735387 and rs1428) were selected and explored for their correlations with the risk of HAPE in the Chinese population. Agena MassARRAY Assay Design 3.0 software (https://seqpws1.agenabio.com/AssayDesignerSuite.html) was used to design the primers (Supplementary Table 1). Five milliliters of peripheral venous blood was collected according to standard procedures in an EDTA-containing anticoagulation tube and stored in an ultralow-temperature freezer for subsequent DNA extraction. The extraction of DNA from whole blood samples was performed using the GoldMag–Mini Purification Kit (GoldMag Co, Ltd, Xi’an, China). Quality monitoring, including concentration and purity assessment, of the extracted DNA was conducted using a spectrophotometer. Finally, genotyping of the six polymorphisms of the *MIR17HG* gene was performed using the Agena MassARRAY RS1000 system according to the manufacturer’s. The Agena Typer 4.0 software (Agena, San Diego, CA, USA) was used to analyze the experimental results and obtain the genotyping data following a previously described method^4,33^.

### Bioinformatics analysis

The HaploReg v4.1 (https://pubs.broadinstitute.org/mammals/haploreg/haploreg.php) database is often used to annotate noncoding genomes on haplotype blocks, including disease-related SNPs. In this study, HaploReg v4.1 was used to predict the potential functional role of candidate SNPs.

### Meta-analysis

This study obtained genotyping data of six SNPs (rs75267932, rs72640334, rs7336610, rs7318578, rs17735387, rs1428) on the *MIR17HG* of the control group from the 1000 Genome Projects (http://www.internationalgenome.org/). The genotyping data of the six SNPs in the case group was obtained through the interpolation procedure. The data obtained above was analyzed by STATA 11.0 software (Stata Corp., College Station, TX, USA)^34^.

### Statistical analysis

The analysis of the general characteristics of the subjects, the chi-square test was used to compare the categorical variable between the case group and the control group. The t-test was used to compare the continuous variable (age) between the case and control groups. The goodness of fit chi-square test was used to calculate the HWE of each polymorphism in the control group to test the representativeness and randomness of the study population. The chi-square test was used to detect the distribution differences of genotype and allele frequency of each polymorphism between the case group and the control group. Logistic regression was used to calculate OR values and 95% CIs to evaluate the strength of the association between each genotype and HAPE by PLINK software. All statistical tests were bilateral, with a significance threshold of 0.05. In this study, the minimum sample size and proportions of the experimental group and the control group were determined by G * Power 3.1.9.2 software^35^. The analysis results indicated that the total sample size should be greater than 388 and that the sample size of each of the control group and case group should be greater than 194. The total sample size in this study was 487, with 243 subjects in the control group and 244 in the case group thus, the sample sizes of this study met the statistical requirements. In addition, the statistical power of this study to detect an effect at the specified level of significance was 99.98%. Multifactor dimensionality reduction (MDR) analysis was performed using MDR_3.0.2 software (http://sourceforge.net/projects/mdr/) to determine a high-order interaction model for the risk of HAPE^36^.

### Ethics approval and consent to participate

This study was approved by the Ethics Committee of the Affiliated Hospital of Qinghai University and was conducted in compliance with the Declaration of Helsinki. The purpose of this study was well conveyed to all participants, and written informed consent was obtained from all participants prior to biological material collection in this study. All subsequent study analyses were conducted in accordance with the approved guidelines and regulations.

### Consent for publication

Written informed consent was obtained from the patients for publication of this report.

## Supplementary Information

Supplementary Information.

## Data Availability

The datasets used or analyzed during the current study are available from the corresponding author on reasonable request.

## Abbreviations

HAPE: High-altitude pulmonary edema
AMS: Acute mountain sickness
SNP: Single nucleotide polymorphism
HWE: Hardy–Weinberg equilibrium
OR: Odds ratio
95% CI: 95% confidence intervals
CT: Computed tomography
MAF: Minor allele frequency
MM: Multiple myeloma
*MIR17HG*: MiR-17-92 cluster host gene
LD: Linkage disequilibrium
