# Supplementary material for: MIR17HG polymorphisms contribute to high-altitude pulmonary edema susceptibility in the Chinese population
Source: Sci Rep. 2022 Mar 14;12:4346. doi: 10.1038/s41598-022-06944-8 (PMC8921515; doi:10.1038/s41598-022-06944-8)
Supplement: Supplementary file 1 — Supplementary Information. [file 41598_2022_6944_MOESM1_ESM.docx]

**Supplementary table 1** Primer Sequences of *MIR17HG* Gene SNPs

| SNP_ID | 2nd-PCRP | 1st-PCRP | UEP_SEQ |
| --- | --- | --- | --- |
| rs75267932 | ACGTTGGATGTTAGAGAGAATGCCGCTCTG | ACGTTGGATGCCCAACCCTAAATTCCATGC | cccCCGCTCTGTTTAAAGCAATGTGTA |
| rs72640334 | ACGTTGGATGGCTTAAGAACTCTGCTAATG | ACGTTGGATGCTATCATTCTGGAGTTGATG | CACTGTTCATTTCACATCCT |
| rs7336610 | ACGTTGGATGACAGCGTTTCACCATGTCGG | ACGTTGGATGAAAAAGTTCCGGCTGGACAC | CTCCTGACCTCAGGTAATCC |
| rs7318578 | ACGTTGGATGGAAATCATCCAGCAGGCTTC | ACGTTGGATGCAGCATGGTCTGGTAGTTTG | TCCTATCACACTGTTCCA |
| rs17735387 | ACGTTGGATGGCTTTCTTTCCAAATATAGGC | ACGTTGGATGAGCCTTAACTATTTGGAGGG | AATAGAAAGTTGTACATGCAAA |
| rs1428 | ACGTTGGATGTCAATATTCTCGTTCTGGAC | ACGTTGGATGTGGCTGTTTGAGTTCTAGCG | tACAATTTCTTAACAGCTTTAAAAT |

**Supplementary table 2**. Stratification analysis by age for the effect of *MIR17HG* SNPs on HAPE risk.

| SNP | Model | Genotype | ≤ 32 | | > 32 | |
| --- | --- | --- | --- | --- | --- | --- |
|  |  |  | OR (95% CI) | *p* | OR (95% CI) | *p* |
| rs75267932 | Genotype | AA | 1 |  | 1 |  |
|  |  | AG | 0.88(0.49-1.57) | 0.657 | 1.42(0.74-2.71) | 0.295 |
|  |  | GG | - | 0.998 | 0.51(0.05-5.79) | 0.589 |
|  | dominant | GG/AG+AA | 0.9(0.5-1.6) | 0.712 | 1.34(0.71-2.52) | 0.371 |
|  | recessive | GG+AG/AA | - | 0.999 | 0.47(0.04-5.3) | 0.542 |
|  | log-additive | / | 0.93(0.53-1.64) | 0.796 | 1.22(0.68-2.18) | 0.508 |
| rs72640334 | Genotype | CC | 1 |  | 1 |  |
|  |  | AC | 1.16(0.63-2.15) | 0.632 | 0.75(0.36-1.54) | 0.43 |
|  |  | AA | 1.61(0.26-10.11) | 0.611 | - | 0.999 |
|  | dominant | AA/AC+CC | 1.2(0.66-2.16) | 0.556 | 0.71(0.34-1.45) | 0.344 |
|  | recessive | AA+AC/CC | 1.56(0.25-9.78) | 0.633 | - | 0.999 |
|  | log-additive | / | 1.19(0.71-2.02) | 0.51 | 0.68(0.34-1.36) | 0.279 |
| rs7336610 | Genotype | TT | 1 |  | 1 |  |
|  |  | TC | 1.37(0.77-2.45) | 0.283 | 1.28(0.66-2.49) | 0.469 |
|  |  | CC | 0.94(0.47-1.88) | 0.87 | 0.71(0.31-1.66) | 0.436 |
|  | dominant | CC/TC+TT | 1.22(0.71-2.1) | 0.476 | 1.12(0.59-2.13) | 0.735 |
|  | recessive | CC+TC/TT | 0.77(0.43-1.39) | 0.388 | 0.6(0.3-1.18) | 0.136 |
|  | log-additive | / | 0.99(0.7-1.39) | 0.953 | 0.86(0.56-1.31) | 0.479 |
| rs7318578 | Genotype | AA | 1 |  | 1 |  |
|  |  | AC | 0.82(0.49-1.38) | 0.45 | 0.73(0.42-1.27) | 0.259 |
|  |  | CC | 0.59(0.24-1.43) | 0.245 | 0.38(0.12-1.19) | 0.097 |
|  | dominant | CC/AC+AA | 0.77(0.47-1.25) | 0.29 | 0.67(0.39-1.14) | 0.14 |
|  | recessive | CC+AC/AA | 0.64(0.27-1.52) | 0.313 | 0.44(0.15-1.34) | 0.151 |
|  | log-additive | / | 0.79(0.54-1.15) | 0.212 | 0.67(0.43-1.04) | 0.074 |
| rs17735387 | Genotype | GG | 1 |  | 1 |  |
|  |  | AG | 1.5(0.87-2.59) | 0.14 | 1.74(0.95-3.17) | 0.071 |
|  |  | AA | 0.8(0.18-3.5) | 0.767 | 0.82(0.18-3.81) | 0.802 |
|  | dominant | AA/AG+GG | 1.42(0.84-2.39) | 0.19 | 1.61(0.91-2.87) | 0.104 |
|  | recessive | AA+AG/GG | 0.71(0.16-3.06) | 0.643 | 0.69(0.15-3.18) | 0.638 |
|  | log-additive | / | 1.26(0.8-1.98) | 0.321 | 1.38(0.83-2.27) | 0.212 |
| rs1428 | Genotype | AA | 1 |  | 1 |  |
|  |  | AC | 1.36(0.76-2.42) | 0.305 | 1.18(0.61-2.29) | 0.622 |
|  |  | CC | 0.98(0.49-1.94) | 0.947 | 0.77(0.34-1.75) | 0.532 |
|  | dominant | CC/AC+AA | 1.22(0.71-2.1) | 0.476 | 1.06(0.56-2.01) | 0.855 |
|  | recessive | CC+AC/AA | 0.81(0.45-1.44) | 0.469 | 0.68(0.35-1.32) | 0.258 |
|  | log-additive | / | 1.00(0.71-1.41) | 0.984 | 0.88(0.59-1.33) | 0.558 |

SNP, single nucleotide polymorphism; OR, odds ratio; 95% CI, 95% confidence interval.

*p* values were calculated by Fisher's exact test or logistic regression analysis with adjustments for age.

Bold indicated that *p* < 0.05 meant the data was statistically significant.

**Supplementary Table 3**. Meta analyzed the impact of MIR17HG polymorphism on the risk of HAPE under the allelic model.

| SNP | Alleles | Case count | Control count | OR (95%CI) | *p* |
| --- | --- | --- | --- | --- | --- |
| rs75267932 | G/A | 63/561 | 72/620 | 0.97(0.68-1.38) | 0.854 |
| rs72640334 | A/C | 61/563 | 73/619 | 0.92(0.64-1.32) | 0.643 |
| rs7336610 | C/T | 293/331 | 345/347 | 0.89(0.72-1.11) | 0.293 |
| rs7318578 | A/C | 158/466 | 211/481 | 0.77(0.61-0.98) | **0.037** |
| rs17735387 | A/G | 125/499 | 129/563 | 1.09(0.83-1.44) | 0.523 |
| rs1428 | C/A | 301/319 | 345/347 | 0.95(0.76-1.18) | 0.636 |

SNP, single nucleotide polymorphism; OR, odds ratio; 95% CI, 95% confidence interval.

Bold indicated that *p* < 0.05 meant the data was statistically significant.
